# Genetic susceptibility to arsenic-induced skin lesions and health effects: a review

**DOI:** 10.1186/s41021-015-0023-7

**Published:** 2015-11-01

**Authors:** Somnath Paul, Sangita Majumdar, Ashok K. Giri

**Affiliations:** Molecular and Human Genetics Division, CSIR-Indian Institute of Chemical Biology, 4, Raja S. C. Mullick Road, Kolkata, 700032 West Bengal India

**Keywords:** Arsenic, Toxicity, Single nucleotide polymorphism, Genetic susceptibility, Epimutagenesis, West Bengal

## Abstract

Arsenic toxicity in humans manifests several outcomes in humans, which include arsenic-induced genomic instability, DNA damage, impaired DNA repair, carcinogenesis, dermatological lesions and other health related problems. Of the 137 million individuals affected, nearly 26 million individuals are in the state of West Bengal, India. Studies have identified dermatological lesions like keratosis, basal cell carcinoma, Bowen’s diseases, squamous cell carcinoma, etc., as key indicators of aggressive arsenic toxicity in humans. Although a large number of individuals are exposed to arsenic but only about 15 to 20 % individuals showed arsenic induced skin lesions. This clearly indicates that genetic susceptibility plays an important role in arsenic susceptibility. Analyses of genetic susceptibility have been carried out to study the prevalence of single nucleotide polymorphisms (SNPs) in number of genes as they might be involved arsenic metabolism and detoxification. It has been observed that a number SNPs in these genes were significantly associated with arsenic induced skin lesions and other health effects. In the present review we try to coalesce the different observations and associations of SNPs with arsenic-induced toxicity, with special emphasis on the study population from West Bengal. We have adopted certain candidate gene approaches to evaluate the association of arsenic-induced toxic outcomes like skin lesions, conjunctival irritations, DNA damage, epimutagenesis, cancer, etc. This review shall be helpful in understanding the importance of genetic make-up of an individual towards evaluating the xenotoxic outcomes, like those in case of arsenic exposure.

## Introduction

A global concern, a potent carcinogen and toxic upon chronic exposure, arsenic-induced toxicity in humans is multi-pronged; having a varied spectrum of patho-physiological outcomes. Nearly 137 million individuals are affected by arsenic in nearly 70 nations all over the world that includes India, Bangladesh, Taiwan, Japan, Chile and parts of China and USA [1, 2]. Apart from geogenic outcomes [3]; industrial and commercial activities have also lead to spread of arsenic like those of “the Toruku Mine incidence” and “the Nakajo-Machi incidence” in Japan in the early twentieth century [4]. Some of the major toxic outcomes of arsenic in humans include oxidative DNA damage, dermatological lesions in form of keratosis, peripheral neuropathy, gastro-intestinal inflammation and cancers of various types like skin, lungs, bladder, liver, etc. [5–9].

**Fig. 1:**
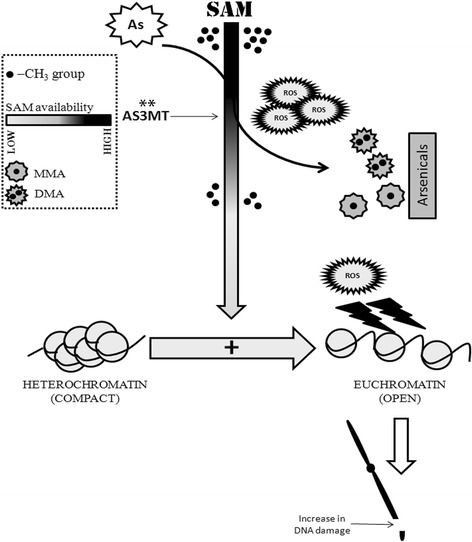
Metabolic events upon arsenic consumption deplete the methylation pool within the cell. “**” indicates that AS3MT is a highly polymorphic enzyme. It’s polymorphic profile can determine the degree of biotransformation of inorganic arsenic within the cellular system. This may induce indirectly the epigenetic susceptibility as discussed in section Polymorphism and arsenic-induced epigenetic susceptibility

Several mechanisms of arsenic-induced toxicity have been proposed and researched all over the world. Of these, enhanced toxicity due to reactive oxygen species (ROS) have been evaluated by several studies which was associated with a plethora of toxic outcomes like arsenic-induced cytogenetic damage, inflammation and carcinogenesis [10–12]. This aggressively oxidizes several cellular components and has been well characterized upon arsenic exposure like oxidative DNA damage [8, 13]. Among the most recent concepts of research, arsenic-induced epigenetic alterations have also been associated with several molecular outcomes of arsenic toxicity. Arsenic biotransformation and metabolism within the cells involves a cascade of enzymes that converts inorganic arsenic to it’s methylated species, using S-Adenosyl Methionine (SAM) as a substrate. This depelete indigenous SAM pool within the cells leading to arsenic-induced global DNA hypomethylation, leading to carcinogenic outcomes by aberrant gene expressions within the cell [14]. It was reported that Myc. overexpression was associated upon arsenic-induced malignant transformation in nude mice [15]. MYC, has a strong association with hepatic and pulmonary cancer with relevant studies showing association with chronic arsenic exposure with liver cancer and lung cancers [16–18]. This may be mediated by arsenic-induced epigenetic alterations of Myc. expression. Also, Myc has the ability to recruit TIP60 (a histone acetyl transferase; HAT) to the chromatin [19]. HAT category of enzymes are important regulators of histone acetylation; responsible for “opening up” of the nucleosomes and increase the accessibility of the transcription factors. This facilitates the transcription, along with DNA hypomethylation. This “opening up” may also enhance accessibility of ROS; leading to an increased degree of oxidative DNA damage. A probable mechanism is elaborated in Fig. 1.

Genetic susceptibility has been one of main proponent of arsenic-induced toxicity. Several population surveys all over the world have associated single nucleotide polymorphism (SNP) with arsenic-induced carcinogenesis. Dermatological lesions in form of raindrop hypo-pigmentation, palmer and plantar keratosis, Bowen’s disease as well as squamous cell carcinoma (SCC) and basal cell carcinoma (BCC) have been considered as hallmarks of arsenic-induced toxicity or arsenicosis [20–22]. Thus, several epidemiological studies have evaluated the association between the dermatological lesions and cancer upon chronic arsenic exposure all over the world. SNP analysis of 594 arsenic-induced dermatological cases found a significant association between arsenic metabolic pathway genes with risk of premalignant skin lesions [23]. Arsenic (III) methyltransferase (AS3MT) is one of the main mediators of arsenic biotransformation. SNP (Met287Thr) of AS3MT has been found to be associated significantly with arsenic-induced skin premalignant lesions [24]. This study was conducted in Mexico, where the participants were subjected to nearly 110 μg/L arsenic through drinking water. The present review is on similar SNP studies conducted in the state of West Bengal, India where nearly 26 million individuals are consuming arsenic through drinking, above the limit of 10 μg/L as prescribed by World Health Organization [25]. In the present review we shall elaborate on the association of genetic susceptibility in arsenic exposed population mainly from West Bengal with significant emphasis on dermatological lesions and cancers.

### Candidate genes responsible for arsenic toxicity in humans

#### Arsenic biotransformation pathway

Arsenic biotransformation is a multistep process involving several enzymes. Some of the notable enzymes like AS3MT (methylated inorganic arsenic), purine nucleotide phosphorylase (PNP; acts as arsenic reductase), glutathione-S-transferase omega (GSTO; reduce arsenic metabolites), etc. have been associated with arsenic biotransformation within the body. Biotransformation of arsenic within the body leads to conversion of arsenic species as well as into organic intermediates for excretion of arsenic through urine. In a study involving more than 200 arsenic exposed samples from the arsenic affected districts of the state of West Bengal, elaborated that among *AS3MT*, *PNP* and *GSTO(1/2)*, only exonic SNP of *PNP* showed a significant association in developing arsenic-induced dermatological lesions [26]. The three exonic SNP of *PNP* associated with arsenic-induced toxicity from this study mostly yielded a plausible condition of structural misnomer orientations of the proteins. For example the Gly51Ser alteration was predicted to alter the charge distribution within the region, which was important since the substitution was close to the arsenic binding site. It was suggested that the degree of arsenic transmethylation and conversion to MMA or DMA determines the susceptibility towards dermatological lesions [24]. As an explanation, the authors of these studies predicted the non-toxic values of DMA compared to MMA and hence a high MMA:DMA within the system pre-disposes an individual towards dermotological lesions [23, 27]. A recent study elaborated that the rs9527 transcript variant of the 10q24.32 (associated with *AS3MT*) in individuals led to a lowering in the quantitative presence of DMA and had a higher risk in developing skin lesions [28]. Thus, genotype of an individual is a significant determinant towards the risk of developing arsenic-induced dermatological lesions.

#### Inflammation and arsenic toxicity

*In vitro* and *in vivo* studies have demonstrated that arsenic-induced toxic effects includes exaggerated expression of several pro-inflammatory as well as inflammatory factors like tumor necrosis factor alpha (TNF-α) and interleukins (IL) like IL6, IL8 [7, 29, 30]. Two studies conducted by our group have found significant association between SNP of *TNF-α* (308 G > A), *IL10* (3575 T > A) and *NLRP2* (rs1043673) with arsenic-induced toxic outcomes in the population from West Bengal. In one study conducted with 207 arsenic exposed individuals with skin lesions and 190 arsenic exposed individuals without skin lesions, it was found that SNPs of *TNF-α* and *IL10* had a higher association towards developing dermatological lesions [31]. Interestingly, since both the SNPs were located in the promoter regions of the corresponding genes, it was found that TNF-α A-allele showed a higher expression of the gene while in IL10 A-allele showed a lower production of IL10 in humans. Hence, genotype characteristic of a individual may dictate the course of inflammatory response upon arsenic-induced dermatological lesions. In another of our study, NLR family, pyrin domain containing 2 (*NLRP2*), a major component of the inflammasome complex imparted a higher risk of arsenic-induced dermatological lesions in individuals with the C/C genotype [32]. The study also observed that coherent association of higher cytogenetic damage within arsenic exposed individuals having this *NLRP2* C/C genotype (rs1043673). Earlier we had found a strong correlation between higher cytogenetic damage in arsenic exposed population with dermatological lesions [33].

#### Polymorphic DNA repair genes

DNA damage and subsequent repair equilibrium within the cell is an important perspective towards cell survival. ROS dependent DNA damage oversees several patho-physiological outcomes in humans including cancers, as elaborated by several authors [34, 35]. Studies have identified involvement of p53-dependent repair and cell regulatory pathways to play an active role in DNA damage recognition; bypassing which leads to development of oncogenic outcomes [36, 37]. Since arsenic consumption generates ROS, concomitant polymorphisms in several DNA repair pathway genes have been associated with increased cytogenetic damage upon arsenic exposure. Analysis of the SNP of *ERCC2* (excision repair cross-complementation group 2) codon 751 (A > C; rs13181), it was found that in case of arsenic-induced hyperkeratotic individuals, an over-representation of A/A genotype was present [38]. There was a decreased degree of DNA repair capacity exhibited by this polymorphic *ERCC2* with A/A genotype [39]. This explains a higher cytogenetic damage observed in arsenic exposed individuals with ERCC2 A/A genotypes [38]. Evaluation of *XRCC3* (X-Ray repair complementing defective repair in Chinese hamster cells 3), rs861539 implied that the distribution of T/T or C/T provides a beneficial protective role towards development of arsenic-induced skin lesions as well as DNA damage [40].

#### Polymorphism of tumor suppressor gene- TP53

The tumor suppressor protein *TP53* or p53 plays a central role in mediating stress and DNA damage responses, leading to either growth arrest for DNA repairing or apoptosis [41]. The close association between codon 72 polymorphism of p53 with skin cancer has been reported by epidemiological survey [42]. In our study population, having chronic exposure to arsenic, a significant association been homozygous arginine at the codon 72 of p53 with arsenic induced keratosis [43]. Since, p53 dependent DNA repair is another mechanistic activity found in humans, it was also found that this polymorphism had a significant increase in accumulation of chromosomal aberrations (CA) among the keratotic individuals [44]. We conducted a study to compare the frequency distribution of chromosomal aberrations (% aberrant cell and CA/cell) between the risk genotype (arginine homozygous) and the reference genotypes (arginine/proline heterozygous and proline homozygous combined) at p53 codon 72 locus with individuals without arsenic-induced skin lesions, individuals with keratosis and total population (two groups combined), and we found that the risk genotype containing homozygous arginine (R/R) had shown significantly higher chromosomal aberrations both in form of % aberrant cell and CA/cell in two study groups individually.

#### Polymorphism of glutathione S-transferase (GST) super family enzymes

Glutathione S-transferases (GSTs) are a superfamily of enzymes, ubiquitously present and has multiple functionality like carcinogenesis [45, 46]. The mechanistic modality of GST include conjugation of xenobiotic substances with glutathione, induction of other enzymes and proteins within the cellular micro-environment, etc. [47, 48]. In our study population of West Bengal, we evaluated the null variants for GSTM1, GSTP1 and GSTT1, where we found a significant association of the GSTM1 null variants with arsenic exposed individuals without skin lesions, indicating of a protective role of GSTM1 null towards incidence of arsenic induced dermatological lesions [49]. This observation was interesting, especially with the fact that enzyme super-families having sequence homology more than 40 %, may have functional compensatory mechanism among the sub-groups of the family [50, 51]. In case of GSTM1, a recent study has evaluated the functional similarity between GSTM1 and GSTM2 *in vitro*, whereby GSTM2 showed an equivalent functional activity with GSTM1 [52]. Thus, although our earlier work observed the protective role of GSTM1 null towards developing arsenic induced skin lesions; the possibility of compensatory mechanism might be of significant importance in ultimately determining the genetic susceptibility towards arsenic induced skin lesions. Although it still remains a question that may be explored further, whether glutathione conjugation nature executed by GSTM2 is 100 % same as that of GSTM1. Thus, this may explain the variability in “protection” upon GSTM1 null genotype.

Taking the arsenic exposed population in West Bengal, India, we hitherto try to put forward the major genes that may be considered as candidate genes to determine the extent of arsenic toxicity in humans, as briefed in Table 1, along with some similar works in other populations.

**Table 1:** Summary of SNP analysis in candidate genes

Gene (Reference)^b^	Genotype; OR (95 % CI)	*p*-Value	Comments	Country (Reference)	Genotype; OR (95 % CI)	*p*-Value	Comments
In West Bengal				Outside West Bengal/India			
*ERCC2* [38]	*codon 751 A > C* (A/C + C/C)^a^ vs A/A; 4.77(2.75-8.23)	<0.0001	The A/A variant (Lys/Lys) of ERCC2 demonstrated a suboptimal level of DNA repair and were significantly associated with arsenic-induced hyperkeratosis.	China [65]	*codon 751 A > C* (A/C + C/C)^a^ vs A/A; 2.36 (1.35-4.14)	<0.01	Lys demonstrated increased risk of arsenic induced skin lesions among the Chinese population.
*IL10* [31]	*promoter −3575 T > A* T/T^a^ vs (T/A + A/A); 2.03(1.26-3.28)	<0.01	TNF-α and IL-10 variants were associated with increased skin lesions as well as overexpression and underexpression of the factors respectively.	Bangladesh [66]	*rs3024496 A > G*	<0.05	rs3024996 was significantly associated with arsenic induced skin lesions after multiple comparison adjustments
*NLRP2* [32]	*codon 1052* (C > A); 0.67 (0.46-0.97) C/C^a^ vs (C/A + A/A)	<0.05	Presence of minor allele (A) lead to prominent risk towards development of arsenic-induced skin lesions.	No Other Studies yet found			
*TP53* [43]	*codon 72* (G > C); 2.086 (1.318-3.299)	<0.001	Arginine homozygous at codon 72 of p53 showed increased risk to keratosis and higher chromosomal aberration in arsenic-exposed individuals.	Taiwan [67]	*codon 72 (G > C)*	<0.01	Proline homozygous or heterozygous showed a relative higher risk in renal cell carcinoma, upon arsenic exposure.
*PNP* [26]	*codon 20 C > T* C/C^a^ vs (C/T + T/T); 1.69(1.08-2.66)	0.02	*PNP* variants are significantly associated with arsenic induced dermatological lesions	Taiwan [68]	*codon 57 C > T* C/C^a^ vs (C/T + T/T) 1.50 (1.03-2.18)	<0.05	PNP SNP results in a modified and significant risk of carotid artherosclerosis along with either of AS3MT or GSTO1 SNP.
	*codon 51 G > A* G/G^a^ vs (G/A + A/A); 1.66(1.04-2.64)	0.04					
	*codon 57 C > T* C/C^a^ vs (C/T + T/T); 1.67(1.05-2.66)	0.04					
*TNF-a* [31]	*promoter −308 G > A* G/G^a^ vs (G/A+ A/A); 3.04(1.78-5.21)	<0.001	TNF-α and IL-10 variants were associated with increased skin lesions as well as overexpression and underexpression of the factors respectively.	Taiwan [69]	*promoter −308 G > A*	<0.05	G/A + G/G (low iAs) had a higher risk of urothelial carcinoma upon arsenic exposure compared to A/A (high iAs) group. OR: 14.98; 95%CI: 2.63-85.44.
*XRCC3* [40]	*codon 241 C > T* (C/T + T/T)^a^ vs C/C; 0.45(0.30-0.67)	<0.0001	T: Methionine allele showed protective role against dermatological lesions as well as cytogenetic damage.	Hungary, Romania, Slovakia [70]	*codon 241 C > T* C/C^a^ : 1.00 C/T:0.7 (0.54-0.92) T/T:0.54 (0.36-0.8)	<0.01	Associated significantly basal cell carcinoma.

^a^ Referent Group

^b^ West Bengal, India Reference; Mean arsenic content in drinking water from West Bengal study population was in the range of 151.74-194.82 μg/L. The WHO recommendation for arsenic through drinking water is 10 μg/L

## Polymorphism and arsenic-induced epigenetic susceptibility

Epimutagenesis is one of the modern terms, first coined by Holliday [53]. Presently, the term refers to the xenobiotic interactions of the cells, which in turn leads to alteration in the epigenomic profile of the cells. This may include alteration in the DNA methylation, histone post translational modifications and miRNA alterations. Arsenic exposure exhibits global DNA hypomethylation [14, 54]. Long interspersed nuclear element-1 (LINE-1) hypomethylation have been found positively associated with increase in arsenic-induced bladder cancer in women, in New Hampshire, USA [55]. Since SAM is an indigenous product of the cell, it’s depletion by arsenic metabolism have been attributed to such epigenetic alterations. As mentioned earlier some genotypic variants of *AS3MT* or it’s splice variants has preferential tendency of either higher or lower MMA:DMA ratio [28, 56, 57]. Contemporary study has evaluated the possible association of hypomethylated blood DNA with increase in arsenic-induced skin lesions [58]. In humans, arsenic-induced epigenetic alterations is believed to be a primary contender for it’s toxicity whereby both hypermethylated as well as hypomethylated promoters of tumor-suppressor as well as DNA repair genes have been reported from study population located in West Bengal [59, 60].

The involvement of differential methylation potential of AS3MT genotypic transcript variants warrants for further association studies to look into SNP of several other such epigenetically significant enzymes like *DNMT3A* (DNA methyltransferase 3A) at 448A > G has been associated with gastric cancer [61]; the homozygous variant of G/G at 201A > G of *DNMT1* having a lower risk of developing breast cancer [62]. Although these two studies are not in conjunction to arsenic; the DNMT-class of enzymes are important determinants of the genomic methylation in all cells. When analyzed in Argentina, the arsenic exposed study population showed an association of *DNMT1A* SNP (rs16999593) with lowering in DMA% [63]. Thus, when we are considering the gene and environment interactions as in case of arsenic exposure, such genotypic parameters should provide prognostic information about the fate of toxic outcomes in humans. Such seeded variability in methylation pattern also increases the probability to fall within the niche of epimutagenic events, whose variability can bring forth different degrees of diseased outcomes in arsenic exposed individuals [64].

## Conclusion

Arsenic toxicity provides a spectrum of pathophysiological outcomes in humans. Since there is a prominent interaction of this xenobiotic factor with the genes, the genotypic features have been considered vividly to understand the fate of toxic outcomes. The genetic make-up of an individual is hereditary. Hence in West Bengal as well as in other parts of the world, the arsenic exposed population possess a varied degree of cytogenetic damage as well as other clinical symptoms like peripheral neuropathy, respiratory disorders, etc. Taking studies in West Bengal as a comprehensive paradigm of arsenic toxicity, we propose that the genotype of an individual provides the signature of toxic fate upon arsenic exposure in individuals.
